# Rodent control to fight Lassa fever: Evaluation and lessons learned from a 4-year study in Upper Guinea

**DOI:** 10.1371/journal.pntd.0006829

**Published:** 2018-11-06

**Authors:** Almudena Mari Saez, Mory Cherif Haidara, Amara Camara, Fodé Kourouma, Mickaël Sage, N'Faly Magassouba, Elisabeth Fichet-Calvet

**Affiliations:** 1 Global Health and Biosecurity Unit, Robert Koch Institute, Berlin, Germany; 2 Projet des fièvres Hémorragiques en Guinée, Laboratoire de Virologie, Conakry, Guinea; 3 CD Eau Environnement, Maizières, France and Faune Environment Expertise, Besancon, France; 4 Department of Virology, Bernhard Nocht Institute for Tropical Medicine, Hamburg, Germany; University of Geneva Hospitals, SWITZERLAND

## Abstract

Lassa fever is a viral haemorrhagic fever caused by an arenavirus. The disease is endemic in West African countries, including Guinea. The rodents *Mastomys natalensis* and *Mastomys erythroleucus* have been identified as Lassa virus reservoirs in Guinea. In the absence of a vaccine, rodent control and human behavioural changes are the only options to prevent Lassa fever in highly endemic areas. We performed a 4 year intervention based on chemical rodent control, utilizing anticoagulant rodenticides in 3 villages and evaluating the rodent abundance before and after treatment. Three additional villages were investigated as controls. Analyses to assess the effectiveness of the intervention, bait consumption and rodent dynamics were performed. Anthropological investigations accompanied the intervention to integrate local understandings of human–rodent cohabitation and rodent control intervention. Patterns of bait consumption showed a peak at days 5–7 and no consumption at days 28–30. There was no difference between Bromadiolone and Difenacoum bait consumption. The main rodent species found in the houses was *M*. *natalensis*. The abundance of *M*. *natalensis*, as measured by the trapping success, varied between 3.6 and 16.7% before treatment and decreased significantly to 1–2% after treatment. Individuals in treated villages welcomed the intervention and trapping because mice are generally regarded as a nuisance. Immediate benefits from controlling rodents included protection of food and belongings. Before the intervention, local awareness of Lassa fever was non-existent. Despite their appreciation for the intervention, local individuals noted its limits and the need for complementary actions. Our results demonstrate that chemical treatment provides an effective tool to control local rodent populations and can serve as part of an effective, holistic approach combining rodent trapping, use of local rodenticides, environmental hygiene, house repairs and rodent-proof storage. These actions should be developed in collaboration with local stakeholders and communities.

## Author summary

In the absence of a Lassa fever vaccine, rodent control is the primary prevention option. An effective rodent control intervention must understand human behaviour towards the rodent such as: human–rodent interactions, cohabitation, and local rodent control measures. We conducted a rodent control intervention at community level in a Lassa Virus endemic area in Upper Guinea (Guinea) accompanied by an anthropological study on people’s perceptions and recommendations on the intervention. Based on our results we seek to broaden the rodent control intervention by including environmental hygiene, house repairs and rodent-proof storage. Chemical treatment has proven effective for rodent control but other factors involved in human-rodent interactions should also be addressed. Our findings highlight the need for Lassa fever prevention and rodent control initiatives to work in collaboration with communities and undertake a holistic approach towards rodent control.

## Introduction

Lassa fever is a viral haemorrhagic fever caused by an arenavirus, which was first discovered in Nigeria in 1969 [1, 2]. The disease is endemic in West African countries, including Sierra Leone, Liberia, Guinea, southern Mali, northern Cote d’Ivoire and Nigeria [3–6]. Lassa fever recently emerged in Benin and Togo [7–9] and affects between 200,000 and 300,000 people per year with up to 5,000 to 10,000 deaths annually [10]. The mortality rate is low (1–2%) in communities in endemic areas but can be as high as 50% among hospitalized patients during outbreaks [11, 12]. In general, most cases remain asymptomatic [13]. In Guinea, an epidemiological survey in human populations showed a high seroprevalence, up to 40%, in the south of the country near the border with Sierra Leone [14]. Acute cases have been reported in regional hospitals [15].

For many years, the Natal multimammate mouse, *Mastomys natalensis*, was considered to be the sole reservoir of the virus [16, 17]; however, a recent study in Guinea and Nigeria showed that two other species, the Guinea multimammate mouse, *Mastomys erythroleucus*, and the African wood mouse, *Hylomyscus pamfi*, can also serve as reservoirs [18, 19]. Humans can be infected by touching objects contaminated with rodent urine, breathing aerosolized particles, being bitten by rodents or consuming rodents [20–24]. Human-to-human transmission occurs in the community and in health care settings [10, 25, 26].

Treatment options are limited, with Ribavirin given early in the course of disease to improve survival in patients with Lassa fever [27]. In the absence of a vaccine, rodent control and human behavioural changes are currently the only options available to prevent Lassa fever in highly endemic areas. In Sierra Leone in 1983, a team from the United States Centers for Disease Control and Prevention reported an experimental approach to reduce the incidence of Lassa fever by controlling the rodent population [28]. The experiment lasted 5 weeks and was spatially focused in a single township. The authors did not find any change in disease incidence between people living in houses with the intervention (trapping) and those in houses without the intervention. It is probable that the experiment was too short to adequately measure an effect on the disease incidence. Furthermore, treatment of only a few houses within a single village led to a rapid re-infestation from neighbouring houses. In light of these limitations, our aim was to perform rodent control on a larger scale and for a longer duration. To that end, we investigated a dozen villages in Faranah, in rural Upper Guinea, where Lassa Virus (LASV) is widely distributed [29]. Previous studies of rodent dynamics have shown that *M*. *natalensis* rodents aggregate in houses during the dry season and disperse into gardens and surrounding fields in the rainy season, where they forage in cultivated areas before the harvest [30]. We therefore planned rodent control interventions inside houses during the dry season only, which starts in November and finishes in April [30].

Studies on rat poison use and availability in Africa are rare, but some examples of acute poison or anticoagulant use and supply in Tanzania [31], South Africa [32, 33] and Sierra Leone show effectiveness in controlling rodent population for short periods of time. These examples also indicate that people primarily use anticoagulants and acute poisons (zinc phosphide) because they are easily accessible, cheap and less labour intensive than trapping. In some cases, the anti-inflammatory drug Indomethacin [34, 35] may also be used instead of rodenticide. Whatever the substance used, the effects of rodent control are short-lived.

Despite the noise and the loss of crops, people often adjust to living alongside the rodents.

As in other studies on rodent control acceptability in Africa [33] we aimed to assess first the feasibility and acceptability of community rodent control activities before extending to an holistic approach including environmental sanitation, house repair and rodent proof containers.

To evaluate the feasibility of rodent control in the Faranah region, we performed an intervention based on chemical rodent treatment in 3 villages, evaluating the rodent abundance before and after treatment. Three additional villages were investigated as controls for comparison with the treatment villages. This article discusses the rodent diversity, rodent abundance and sociocultural factors affecting the feasibility, efficacy and acceptability of chemical rodent control. Based on our experiences during 4 consecutive years in Upper Guinea, we discuss ideas for advancing sustainable rodent control.

## Materials and methods

### Study sites

Six villages were chosen in the surrounding area of Faranah; 3 to serve as controls and 3 to perform the intervention. The choice of villages was based on their remote location from a paved road, a size not exceeding 1000 inhabitants, less than 45 minutes driving time from Faranah and the presence of LASV (see map in Fichet-Calvet et al. 2016). We first sampled rodents in 10 villages in November-December 2013. Of the 10 villages, 9 were positive, with a range of 1 to 10 LASV-positive *M*. *natalensis* rodents in each village. The villages were classified as either high or low prevalence and therefore allocated randomly to control and treated groups, leading to 3 villages in the control group with the following prevalence rates: 20.6% (7/34) in Sokourala, 19.6% (10/51) in Damania, and 2.1% (1/46) in Sonkonia. The 3 villages in the treated group had the following prevalence rates: 20.0% (8/40) in Dalafilani, 17.8% (8/45) in Yarawalia, and 3.8% (2/52) in Brissa. Thus, two villages with a high prevalence and one village with a low prevalence were included in each category (control versus treatment).

### Treatment

We planned to perform the treatment intervention during the dry season (November-April), when the rodents are expected to aggregate inside. We employed a treatment using anticoagulant rodenticide baits, which we distributed in baiting stations (Coral, Ensystex Europe) to all open houses of the village. Rodent control is more effective when it is managed at the collective level rather than at the individual level [36]. Two baiting stations were distributed in each room, totalling 300–600 stations per village, according to their size.

During the first three years, we purchased the anticoagulants locally available in Conakry. This treatment was a mixture of wheat and Bromadiolone, labelled at 0.01%, sold in small sealed bags (S1 *Fig*). In these baits, Bromadiolone was titrated at 30 ppm, which corresponds to a concentration of 0.003% (V. Lattard, pers. com. according to [37]). The concentration was therefore 3 times lower than that claimed on the label. During the last year, we used Difenacoum at 0.005% mixed with cereals and paraffin as bait in cubes weighing 50 g (Rodenthor bloc, Ensystex Europe). The duration of chemical treatment was 10 days during the first 2 years and 30 days during the last 2 years (S1 *Table*). In the villages with treatment, dead rodents found outside of their burrows were collected by the team and cremated in a special hole outside the village. This hole was also used to burn the contaminated waste produced by necropsies during the routine sampling of rodents for LASV testing. For comparison with the captured rodents, dead rodents were numbered and identified during years 3 and 4.

### Bait consumption

To verify whether rodents were eating the bait, we evaluated the consumption during the whole process of treatment during years 3 and 4. On day 1, the bait stations were weighed empty and filled with 50 g of bait before being set. On day 2, each station was weighed and the value was recorded (S2 *Fig*). The difference between day 1 and day 2 indicated the daily consumption for each bait station. The values were thereafter summed for all the stations set in the village.

Checking and baiting were performed each day during the first 5 days and then every 2 or 3 days between days 6 and 30. The reason for the more frequent checking at the beginning and less frequent checking at the end of the process was the decrease in the local rodent population due to the anticoagulant rodenticide activity. Typically, the highest rodent mortality occurs between 3 and 10 days after the first anticoagulant ingestion [38].

### Rodent abundance

To measure the local rodent abundance, we set 120 traps along a transect crossing the villages. Two Sherman live traps (Sherman Live Trap Co., Tallahassee, FL, USA) were set per room over 3 consecutive nights. The traps were checked by team members each morning, and the captured animals were immediately necropsied in situ according to a BSL3 procedure [39, 40]. The animals were morphologically identified, and several biopsies were collected for further ecological and virological analysis. Morphological identification was facilitated from knowledge gained during 14 years of work with small mammal species in this area. Previous studies using both morphological and molecular identifications in the same geographical area have shown that *M*. *natalensis* was predominantly living in houses while *M*. *erythroleucus* was never found in houses [41].

The abundance was estimated by using the trapping success for each 3-night trapping session (Σ trapped rodents during 3 days/360 trap-nights x 100). A trapping session was performed twice in each treated village, i.e., before and after treatment, and once in the control villages. In total, we analysed 14,394 trap nights. Data are available in DOI 10.6084/m9.figshare.5545267.

### Anthropological investigations

We applied several techniques to collect local perception regarding cohabitation with rodents and the intervention after the first treatment and throughout the 4 years. Qualitative investigations included focus group discussions with groups of women and men separately in each village (11), in-depth interviews (39), informal discussions and participant observation and photographs.

Focus group discussions are used to collect views on a particular topic from specific groups of people with similar experiences. These discussions usually include approximately ten individuals and two moderators, one who asks questions and stimulates the discussion and another who takes notes. We used this technique to explore peoples’ views on the project activities and on why rodents live with them.

In-depth interviews were conducted individually with people who could provide detailed information on the research topic.

We conducted informal discussions with the team and people living in the villages regarding the intervention. Photographs documented the domestic space, the distribution of the houses, and building materials, among other inputs. In addition, we distributed a short quantitative questionnaire in August 2016 to evaluate the acceptance of our project by the villagers. We selected people in each village according to their availability, knowledge and willingness to participate [42]. Oral consent was obtained before distribution of the questionnaire. In total, 203 people were questioned regarding their views of the project and specifically whether rodents had disappeared from their houses and for how long this absence had persisted.

All the discussions were facilitated by a translator in Malinke, Djallonke and French. We obtained permission from the local health authorities (Directeur Régional de la Santé, Directeur Préfectoral de la Santé) in Faranah and in each village from the local chief, elders and youth leaders before starting the research activities.

#### Ethics statement

Informed consent for discussions and interviews was obtained orally and recorded. Ethical clearance 066/INSP/12 was obtained from the Guinean ethics board.

### Challenges

During the Ebola Virus Disease (EVD) epidemic, lasting from March 2014 to January 2016, we were not able to follow the original planned intervention. People refused the trapping in some villages due to the fear and mistrust generated by the EVD epidemic. In year 2 for example, a trapping delay of 6 months meant that we did not have time to perform 1 trapping session in 6 villages before treatment, 1 trapping session in 6 villages after treatment, nor elimination in 3 villages before the end of dry season in April. Furthermore, 2 of 3 control villages refused to adhere to the experiment. In year 3, one control village was still reluctant to participate. In years 2 to 4, we therefore reduced pre- and post-treatment trapping in treated villages only, and kept a single trapping in control villages where possible. Details are presented in a supplementary document (S1 *Text*).

### Statistical analysis

A comparison of the daily consumption between the Bromadiolone grains and the Difenacoum blocks, and also between villages was evaluated through a linear model (“lm” tool in R software, R Development Core Team, 2017), with the quantity as a response variable, and molecule, village and days as explanatory variables. Rodent dynamics were analysed using the linear mixed effects model (“lme” tool in R software), with the abundance of *M*. *natalensis* as a response variable, and treatment as an explanatory variable coded as a fixed effect. The village and the year were also entered in the model as random variables. The abundance of *M*. *natalensis* was here estimated through the number of trapped animals since the denominator of TS was always the same, i.e. 360 trapping nights. To analyse the possible trend of rodent abundance between year 1 and year 4, a third model was implemented in R, with the abundance of *M*. *natalensis* as the response variable, and type of village (treated *vs* control) and year (year1, year4) as explanatory variables in a 2-ways interaction. To evaluate the relationship between rodent abundance and bait consumption, a simple regression was performed with 6 points (3 villages x 2 years).

## Results

### Bait consumption

**Fig 1:**
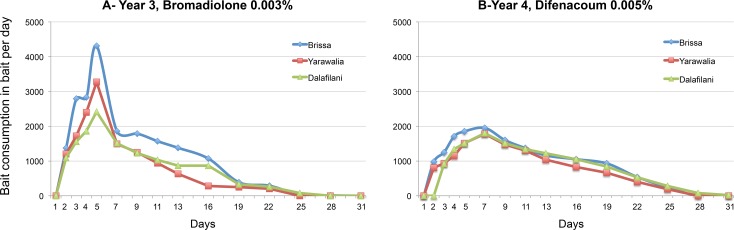
A. Consumption of rodenticide bait per day in year 3 (2015–2016) in the 3 tested villages (Brissa, Yarawalia and Dalafilani) over 30 days (wheat bait mixed with 0.01% Bromadiolone). B. Consumption of rodenticide bait per day in year 4 (2016–2017) in the 3 tested villages (Brissa, Yarawalia and Dalafilani) over 30 days (paraffin blocks mixed with 0.005% Difenacoum).

In both year 3 and year 4, the bait consumption at the beginning of the baiting period was low and then increased, reaching a maximum at day 5 or day 7 depending on the year (*Fig 1*). Subsequently, bait consumption gradually decreased to zero after days 25 or 28, depending on the year.

The bait consumption was similar regardless of the molecule (p = 0.06, df = 72), Bromadiolone (sum = 46.440; median = 0.940; IC95% = 0.723.8–1.340.2 kg) or Difenacoum (sum = 39.140; median = 0.930; IC95% = 0.682–1.057 kg). Village effect was evident, with a bait consumption higher in Brissa, than in Yarawalia (p = 0.007, df = 72, estimate = -287.7) and Dalafilani (p = 0.006, df = 72, estimate = -292.7).

### Rodent abundance

**Fig 2:**
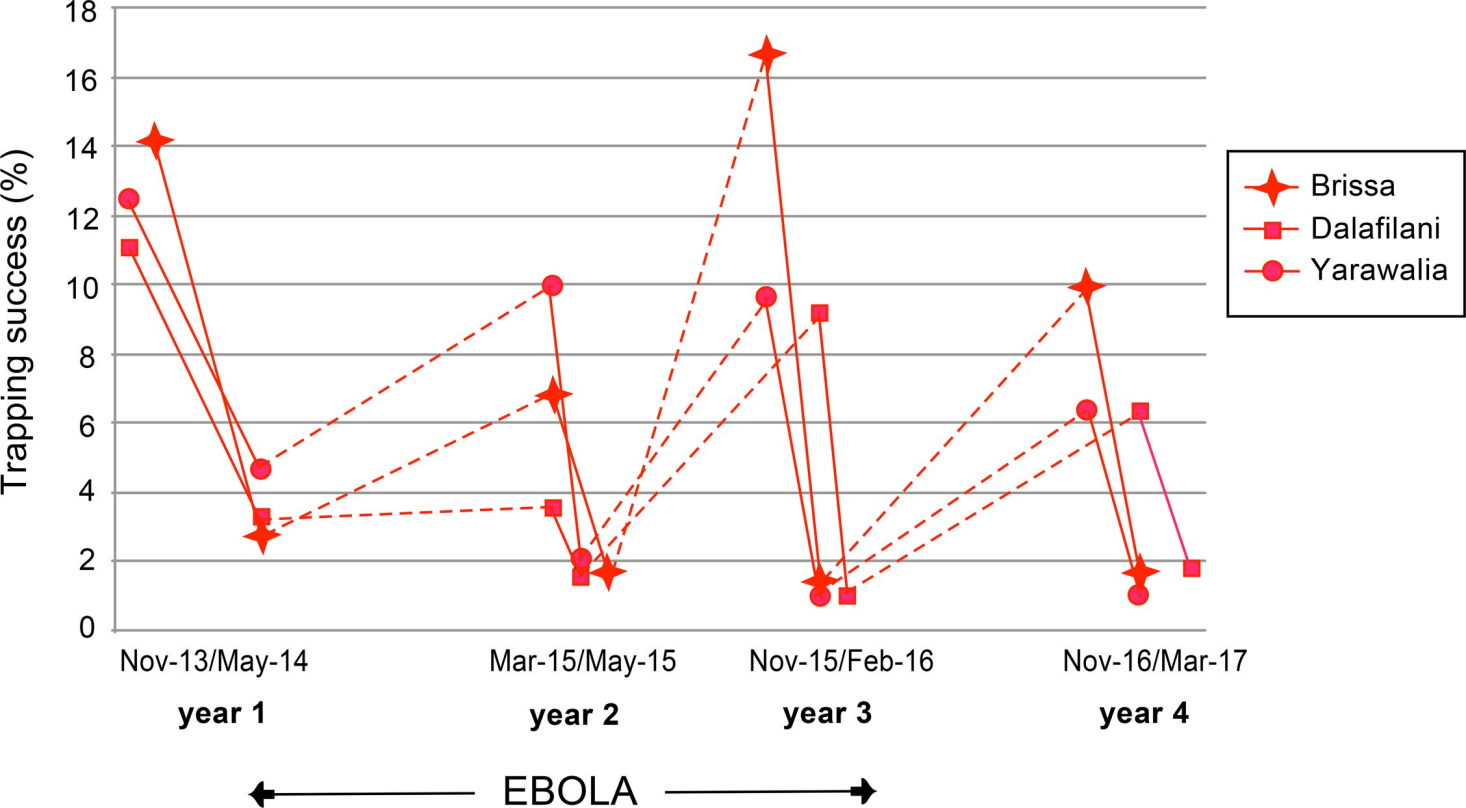
Dynamics of *M*. *natalensis* measured by the trapping success inside houses in 3 villages over 4 years. Solid lines illustrate the crash of the population after treatment, and dashed lines illustrate the population recovering from one year to another. The duration of the Ebola outbreak is shown between the arrows.

The villages in our study were primarily inhabited by *M*. *natalensis*, which represented 94% (1047/1114) of the captured animals (*Table 1*). The dynamics of *M*. *natalensis* population changes in treated villages are shown in *Fig 2*, where the values of trapping success (TS) are plotted according to the time schedule, either before or after treatment.

**Table 1:** Diversity of small mammals trapped inside houses per village (sum over all 4 years). Pink: animals collected after treatment intervention during years 3 and 4.

Species	Brissa		Dalafilani		Yarawalia		Damania	Sokourala	Sonkonia	Total trapping	Total poisoning
*Mastomys erythroleucus*			3							3	0
*Mastomys natalensis*	200	288	137	220	173	145	201	126	210	1047	653
*Nannomys sp*	1	1								1	1
*Praomys daltoni*	6		6	1	20		4	1	4	41	1
*Rattus*	5		4	15	11	5		1		21	20
*Crocidura sp*							1			1	0
Total	212	289	150	236	204	150	206	128	214	1114	675
*% Mastomys natalensis*	94%	100%	91%	93%	85%	97%	98%	98%	98%	94%	97%

**Fig 3:**
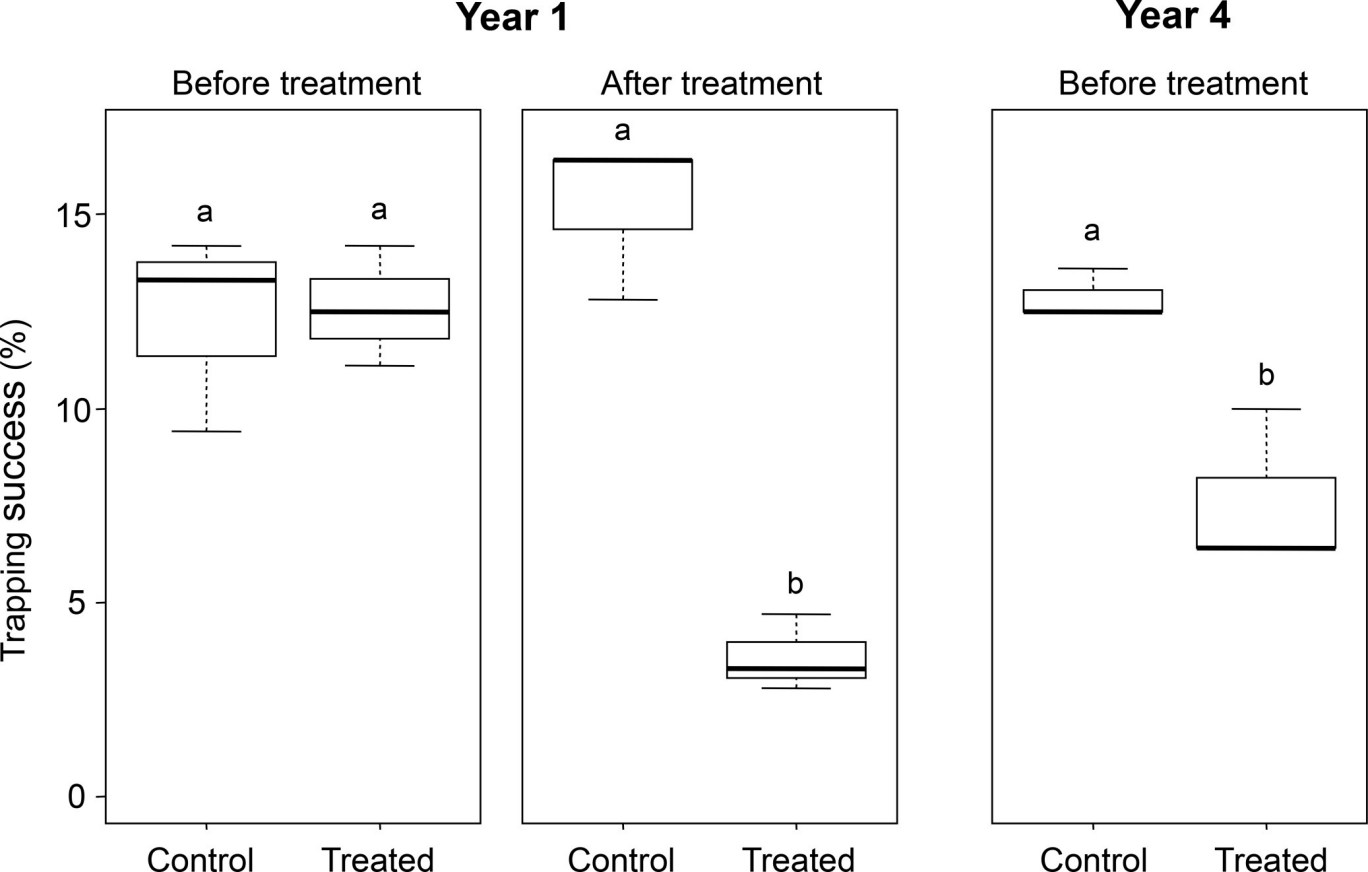
Median trapping success in control and treated villages during years 1 and 4. After treatment, the treated villages had a significant lower abundance than control villages. After 3 years, treated villages had a significant lower abundance than the control villages. Letters “a” and “b” indicate difference of trapping success between sessions (t test, p<0.05).

After treatment, the population was significantly lower than before (p<0.0001, df = 11). After 3 years of treatment, the treated villages had a significant lower abundance than the control villages (significant interaction year 4 x treated villages, p = 0.03, df = 8, estimate = -19.7, in *Fig 3*).

A simple regression between abundance and bait consumption during year 3 and 4 gives a significant coefficient of correlation (r2 = 0.94, df = 4, p = 0.001). The highest abundance observed in Brissa corresponded to the highest bait consumption.

### Housing quality

**Fig 4:**
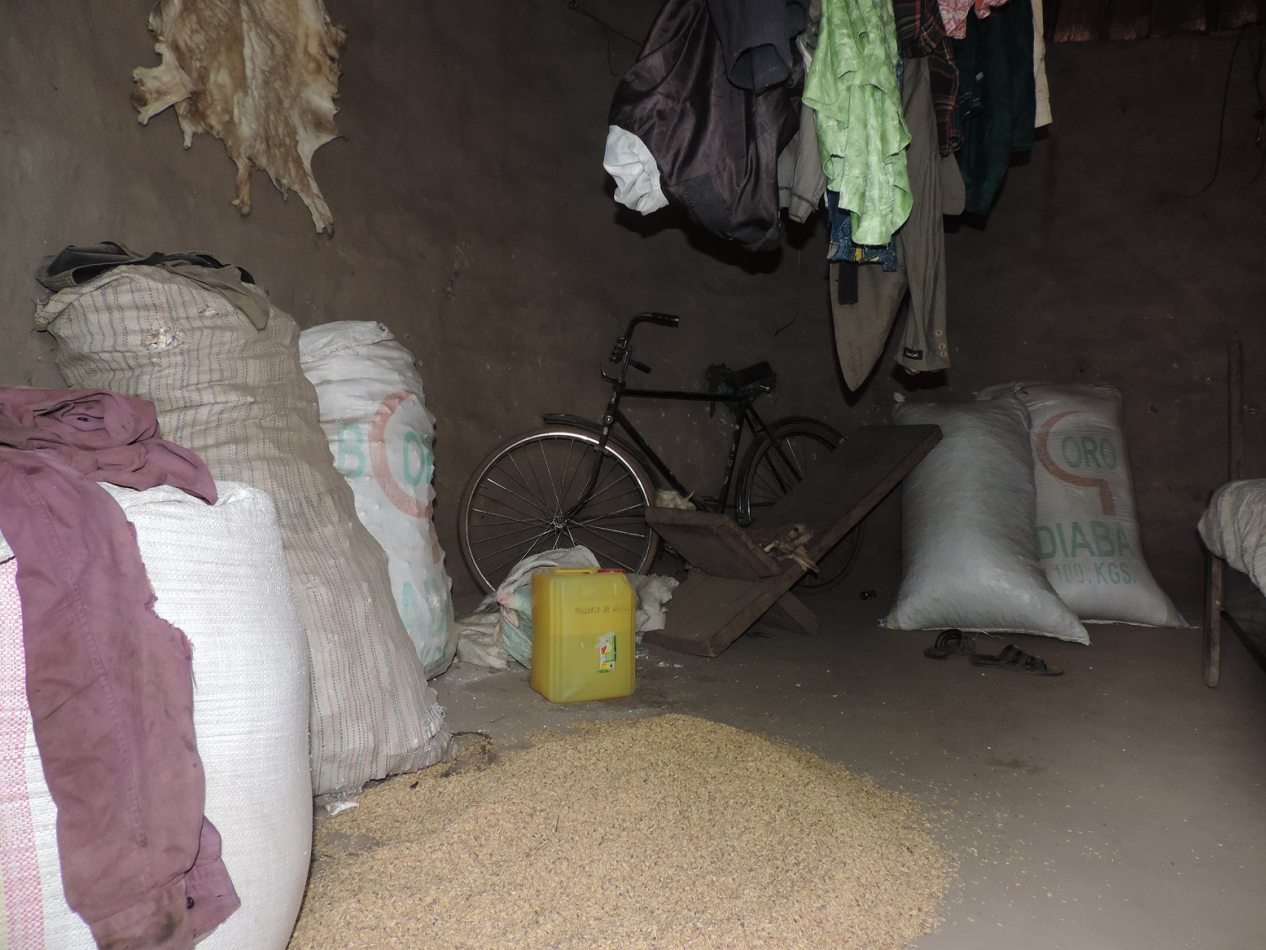
Domestic habitat in study area.

The most frequent building type is a round Sudanese-style mud hut with thatch roof; people use sleeping huts or rooms as storehouses; kitchens are adjacent buildings which follow the same construction style. Owners of concrete and metal-roofed buildings, which have several rooms, typically reserve one room as a store to protect harvest and seeds from fires (*Fig* 4).

### Food storage

Granaries are rare in the region; many people stopped using granaries because they were unable to prevent fires and thefts.

Women can preserve condiments, dry fish and leftover food in wooden boxes. Small quantities of rice remain freely available in rooms, especially in women’s rooms, for weekly family feeding. Before consumption, husk-rice is boiled, dried on the ground several times and pounded. During the drying process, rice can be transferred from the ground outside the house onto the floor inside the house or kitchen several times until the rice is dry.

Rodent-proof containers for post-harvest storage are not available in the region beyond plastic bags and plastic and metallic containers with and without lids, which are also used to store water, local handmade condiments and other crops.

Consequently, in certain villages, people highlighted the need for building food storage and making available rodent-proof containers in parallel with poisoning or trapping.

### Perception of the rodents and their control

Rodents are considered a nuisance because of their effects on food stocks and personal properties. Individuals believed that it would be impossible to kill ‘all’ rodents. For them, they live in rural areas, surrounded by fields and vegetation, and are therefore in permanent cohabitation with rodents. Persons living in the periphery, bordering the bush, complained that rodents come back sooner to these houses than to those in the middle of the village.

Locally, preventive measures taken against rodents are very limited: acute poisons available on the market are used by individual owners in kitchens, stores and rooms before harvesting crops, when people are annoyed by rodent noise or when mice damage their belongings. Several people use Indomethacin, instead of poison because they want to prevent small children and domestic animals from accidental intoxication with poison. Adults may have cats, and the presence of cats was reported to result in fewer rodents in some houses. Children perform rodent trapping by hand when they find a nest in the house or have dogs with them to hunt rodents in the fields.

### Perception of the intervention

#### Acceptability and effectiveness

In all villages, a majority of interviewed persons were exited and receptive about the arrival of a project aimed at killing rodents. Different immediate benefits from controlling rodents were cited, including protection of food and belongings, more peaceful nights in the absence of noise, children not being bitten by mice at night and no introduction of other predators (e.g., snakes) following the rodents into the houses.

People reported in three intervention villages that the product used in the intervention was effective in reducing the number of rodents. Their evaluation of the product’s efficiency was based via visual perception of dead rodents, by the decrease in noise and nuisance, and by indicative odour of decaying mice corpses.

#### Limitations and ways for improvement

In the focus groups conducted after the first treatment, people reported that rodents returned after a few weeks (3 to 4). However, when we questioned the villagers again after year 4, when the treatment lasted for 1 month, people reported that rodents were back in their houses in 1 month (4/32 = 12.5% in Yarawalia), 2 months (18% (8/45) in Dalafilani and 47% (15/32) in Yarawalia), 3 months (100% (33/33) in Brissa, 71% (32/45) in Dalafilani, and 37.5% (12/32) in Yarawalia), or 4 months (11.11% (5/45) in Dalafilani and 3.1% (1/32) in Yarawalia) after treatment.

After the 4 treatments, individuals noted that the treatment required the cooperation of the entire village to be efficient: “If your neighbour is not participating in the treatment, the rodents from his house will come into yours”.

Empty houses are a focus for rodent concentration. In the villages surrounding Faranah, many people leave their village during the dry season to work in the gold mines located in north-eastern Guinea. In one village, approximately 100 out of 800 total villagers left their home for 3–4 months in 2014.

Villagers also suggested that the treatment was not sufficient on its own; other actions such as building community storage structures or the dissemination of hygiene practices were highlighted as complementary for the control of the rodents in their villages.

Hygiene measures such as: cleaning the areas surrounding the house, placing dirty dishes in buckets with water after dinner, washing hands with soap after meals or minimizing hiding and nesting places for rodents were most commonly seen as secondary activities.

#### Keeping momentum

In certain villages, people want to continue with the rodent control despite the end of the project, but they need advice regarding: how and when to use the poison, safely manipulate rodent corpse, clean the surrounding areas of the houses, and organize the accessibility to the rodenticide and rodent proof containers.

Certain individuals also mentioned that after 4 years, people now understand the intervention and have learned that rodents can give them diseases. Residents also mentioned an increase in the awareness of Lassa fever as part of the intervention benefits.

Human factors also contributed to the acceptance of the intervention. The development of trust was an on-going process among the population and project staff, and the long-term intervention was advantageous; people had the opportunity to adhere to the intervention after witnessing from their neighbours that there was no harm. Other good practices noted by residents included that we never had conflicts and that we were regularly present in their villages.

## Discussion

### Bait consumption evolution

The consumption curves of the two compounds, Bromadiolone and Difenacoum, showed that both are effective and that after 28 days, no rodents were feeding. This complete lack of consumption at the end of the operation also showed that there was no resistance phenomenon. A resistance phenomenon is visible when the consumption curve makes a plateau, even peaks again after 15–20 days, due to resistant individuals, which continue to eat the bait. Despite a difference of amplitude between the two curves (Bromadiolone vs Difenacoum), the pattern is similar and quantities of bait were equally eaten at the end of the process when the 3 villages are taken into account. Only one village (Brissa) showed a higher bait consumption, which corresponds to a larger rodent population in years 3 and 4. The peak of Bromadiolone consumption observed during day 4–6 may be due to the behaviour of the rodents, which transport the wheat seeds to their burrows [43]. Paraffin blocks used in year 4 are generally recognized to have a lower palatability and consumption rate than whole cereals. The post-treatment trapping showed that the efficacy was similar between the 2 baits.

### Rodent population evolution

Information collected from villagers indicated that the rodents returned very quickly. Similar observations were done by the population in Sierra Leone after using poison [44]. Increasing the treatment duration to 30 days caused the rodent population, according to local individuals’ observations, to remain low for a longer period of time, with treatment effects persisting for approximately 2 to 3 months. This period would correspond to a return to the carrying capacity of a *M*. *natalensis* population if 90% were initially eliminated (calculation in [45]). Two months correspond also to the mean period for recovery of *R*. *rattus* populations after removal trapping in villages located in Uganda [46]. Assessment of trapping success after treatment was below 2% (years 3 and 4), which is comparable to an elimination performed with continuous trapping during one month [47].

As a consequence, the populations of *M*. *natalensis* oscillated from one year to the next, mimicking seasonal variations. The decline observed in our experiment, occurred however during the dry season when *M*. *natalensis* populations were expected to be abundant indoors, as shown by sampling during Year 1 in control villages after treatment (*Fig 3*) or in longitudinal studies performed in 2 other villages in the same region (Fichet-Calvet et al 2007). In this study, the decline is therefore due to our experiment and not to a normal seasonal effect. A comparison of rodent abundance between year 1 and year 4 showed a slight decrease in the treated villages. This finding needs to be confirmed in a long-term study. Year 2 had a slightly different pattern, as the abundance was surprisingly low before treatment. The trapping before treatment in year 2 was conducted in March, not in November as usually performed. This shift towards the end of the dry season may explain the altered abundance of rodents indoors because they had begun to disperse outdoors [30]. However, the difference might also be due to human behaviour. We observed that villagers in Dalafilani used rodenticides and increased the number of cats between 2 trapping sessions, which may explain the similar trapping success rates measured after treatment in year 1 and before treatment in year 2. This intensification of rodent control by villagers influenced the subsequent rodent abundance.

After 4 years, however, we conclude that once-yearly treatment is not sufficient to maintain the low population abundance of *M*. *natalensis*. There are several reasons for the return of rodents 2 to 3 months after the end of operations: 1) the high proliferation of this species, whose mean litter size is 9.2 (3–14 in [48]; 2) the survival of a few animals in the fields surrounding the houses, which allows recolonization of the human habitat; 3) several closed houses in which we were not able to enter and deposit of the poisoned baits, which may have served as shelters from which the rodents could recolonize houses in the surrounding areas; 4) several very attractive foods available for rodents; 5) the porosity of the walls and roofs, allowing rodents to enter very easily; and 6) the low prevalence of predators, such as cats and dogs. Points 1) and 2) would be more difficult to change because animals’ inherent biological traits are not very easily influenced. Experiments concerning fertility control in rodents are rare because chemosterilization is difficult to practice in the field [49, 50]. The surrounding fields are also the natural optimal habitat for *M*. *natalensis*, and it seems unrealistic to remove them from this habitat. Recent studies on *M*. *natalensis* movements in different microhabitats in the villages confirm the need to expand rodent control measures outside the house to nearby field and gardens [51]. However, points 3) to 6) could be modified more easily in collaboration with the residents taking advantage of the knowledge we are generating together.

### Towards sustainable rodent control

Based on our findings we present in the following lines some ideas for improvement and work towards a sustainable and holistic rodent control intervention.

#### Removal trapping

A first modification should integrate a combination of poisoning with regular trapping. Poisoning would continue to be practised in the dry season, and trapping would take over for the remainder of the year, with consequent training on safe manipulation of dead rodents. Each owner would thus be able to reuse her / his traps. Additional measures linked to the points described above may also be developed.

#### Walls and roofs

In Guinea, human habitations are porous and different rooms serve as stores (sleeping room and kitchen), then rodent exclusion methods must be intensified. One of the greatest challenges faced by rodent control programmes is the capacity to build rodent-proof habitations. According to studies performed in hantavirus [52], leptospirosis [53] and plague [31] endemic areas, rodent exclusion methods include: fixing cracks, applying galvanized wire mesh in openings, replacing broken windows and frames, replacing damaged or inadequately sealing doors, application of draft-sealing strips to doors, and sealing of soffit gaps with small trips of lumber. Most of these methods must be adapted to the architecture in Faranah region and aeration practices. An improvement would be to use the strongest materials for foundations (most burrows are dug at the junction of the wall with the floor) and to have doors and windows with mesh net and that fit without large cracks or gaps.

#### Stocks of food and water

According to the population “even if you kill some rodents, others will arrive to fill the gap in search of food”. This commensality arises from the fact that rodents and humans share the same foods and, as such, have an indivisible relationship. We propose to reduce the attractiveness of foodstuffs by storing them in bags with odour-proof barrier layers. This food-storage system has been successfully tested in Tanzania, where limited rodent damage was recorded, “presumably because the rodent cannot smell the grain inside” [54].

#### Natural predators: Cats and dogs

A recent study on landscapes of fear created by natural predators in Swaziland showed that only the combination of dogs and cats effectively drove away rodents [55].

Additional research is needed to better understand the availability and affordability of cats, or the effect on rodents if a cat is in the house, e.g., whether it eats the rodents or only hunts them and/or prevents them from entering the house, is minimal. Additional research is needed to better understand the capacity of cats to scare off rodents, the relationship between people and cats and dogs, and additional public health problems related to cat ownership.

#### Closed houses

Undertaking rodent control at the village level requires a high commitment from both staff in the field and the local population. People need to open the doors of their houses so that the team can place the bait station and check and collect dead animals and weigh the bait station and re-bait it if necessary on the following days.

Two circumstances affect the treatment: the migration to mining areas and the workload on the farms. In the case of miners, anticoagulants could be placed in their houses before people left the village to reduce the potential that these homes would become refuges used by rodents. In the case of farmers, anticoagulants could be placed early in the morning.

#### Community involvement

We have started exploring ways to address rodent control with the local population and authorities. We need information on the availability and affordability of rat poison in these villages as well as how much money individuals invest annually on poison and the possibilities for re-orienting this individual investment into a joint action at village level. For each village, the cost of anticoagulants was around 200 € per month of treatment, to which was added the initial investment of baiting stations (1000 € per village). This is certainly too high for very poor villagers and only a concerted program between the Ministry of Health and the Ministry of Agriculture would be possible to conduct such programs at a large geographical scale and over a prolonged period of time.

## Conclusion

Based on these findings and the acceptability of rodent control activities at community level, we aim to promote, in coordination with health and agricultural authorities, a more holistic approach [56], including rodent trapping and poisoning, environmental hygiene, personal hygiene, house repairs and rodent-proof storage. The present scenario creates the potential to develop a research-based project and design a collective "one-health" action [57] for rodent management and Lassa fever control.

## Supporting information

S1 Table Treatment protocol in the 3 tested villages over 4 years. (DOCX)

S1 Fig Bag of rodenticides available in Conakry used during the three first years of the study, composed of wheat and labelled at 0.01% Bromadiolone. (TIF)

S2 Fig Weighing of bait during chemical treatment. Photo credit: Mory Cherif Haidara.
(TIF)

S1 Text Ebola interference. (DOCX)
