# Supplementary material for: Rodent control to fight Lassa fever: Evaluation and lessons learned from a 4-year study in Upper Guinea
Source: PLoS Negl Trop Dis. 2018 Nov 6;12(11):e0006829. doi: 10.1371/journal.pntd.0006829 (PMC6219765; doi:10.1371/journal.pntd.0006829)
Supplement: S1 Table — (DOCX) [file pntd.0006829.s001.docx]

**Supplementary information**

**Rodent control to fight Lassa fever: evaluation and lessons learned from a 4-year study in Upper Guinea**

ALMUDENA MARI SAEZ^1^*, MORY CHERIF^2^, AMARA CAMARA^2^, FODÉ KOUROUMA^2^, MICKAEL SAGE^3^, N’FALY MAGASSOUBA^2^, ELISABETH FICHET-CALVET^4^

*1. Robert Koch Institute, Global Health and Biosecurity Unit, Nordufer, 20, 13343 Berlin, Germany; 2. Projet des fièvres Hémorragiques en Guinée, Laboratoire de Virologie, Nongo Conteya, BP 5680, Conakry, Guinea; 3. CD Eau Environnement, 2 rue de Belfays, 70190 Maizières, France; 4. Bernhard Nocht Institute for Tropical Medicine, Department of Virology, Bernhard-Nocht Strasse 74, 20359 Hamburg, Germany*

* mari-saeza@rki.de

Table S1: Treatment protocol in the 3 tested villages over 4 years.

| **N°** | **Year** | **Month** | **Village** | **Duration** | **Compound** | **N stations** |
| --- | --- | --- | --- | --- | --- | --- |
| 1 | 2014 | March | Dalafilani | 10 days | Bromadiolone 0.003% | 594 |
|  | 2014 | March | Yarawalia | 10 days | Bromadiolone 0.003% | 414 |
|  | 2014 | April | Brissa | 10 days | Bromadiolone 0.003% | 604 |
| 2 | 2015 | April | Dalafilani | 10 days | Bromadiolone 0.003% | 600 |
|  | 2015 | April | Yarawalia | 10 days | Bromadiolone 0.003% | 400 |
|  | 2015 | May | Brissa | 10 days | Bromadiolone 0.003% | 480 |
| 3 | 2015 | December | Brissa | 30 days | Bromadiolone 0.003% | 400 |
|  | 2015 | December | Yarawalia | 30 days | Bromadiolone 0.003% | 380 |
|  | 2016 | January | Dalafilani | 30 days | Bromadiolone 0.003% | 400 |
| 4 | 2016 | December | Brissa | 30 days | Difenacoum 0.005% | 420 |
|  | 2016 | December | Yarawalia | 30 days | Difenacoum 0.005% | 310 |
|  | 2017 | January | Dalafilani | 30 days | Difenacoum 0.005% | 400 |
