# Supplementary figures and images for: Rodent control to fight Lassa fever: Evaluation and lessons learned from a 4-year study in Upper Guinea
Source: PLoS Negl Trop Dis. 2018 Nov 6;12(11):e0006829. doi: 10.1371/journal.pntd.0006829 (PMC6219765; doi:10.1371/journal.pntd.0006829)

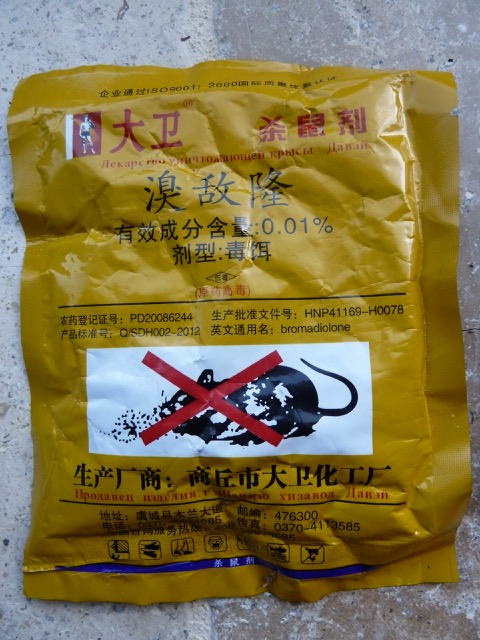

Supplement: S1 Fig — (TIF) [file pntd.0006829.s002.tif]

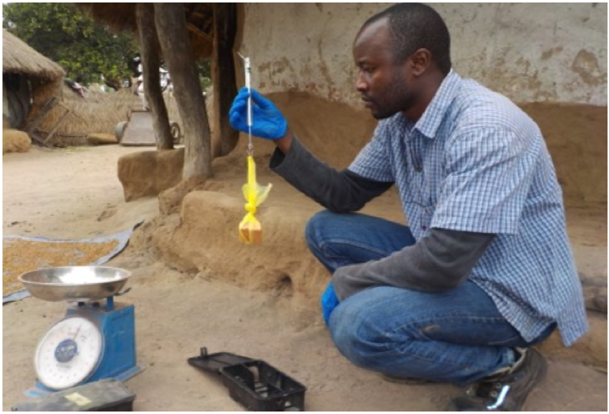

Supplement: S2 Fig — Photo credit: Mory Cherif Haidara. (TIF) [file pntd.0006829.s003.tif]
