# Supplementary material for: Rodent control to fight Lassa fever: Evaluation and lessons learned from a 4-year study in Upper Guinea
Source: PLoS Negl Trop Dis. 2018 Nov 6;12(11):e0006829. doi: 10.1371/journal.pntd.0006829 (PMC6219765; doi:10.1371/journal.pntd.0006829)
Supplement: S1 Text — (DOCX) [file pntd.0006829.s004.docx]

**Supplementary information**

**Rodent control to fight Lassa fever: evaluation and lessons learned from a 4-year study in Upper Guinea**

ALMUDENA MARI SAEZ^1^*, MORY CHERIF^2^, AMARA CAMARA^2^, FODÉ KOUROUMA^2^, MICKAEL SAGE^3^, N’FALY MAGASSOUBA^2^, ELISABETH FICHET-CALVET^4^

*1. Robert Koch Institute, Global Health and Biosecurity Unit, Nordufer, 20, 13343 Berlin, Germany; 2. Projet des fièvres Hémorragiques en Guinée, Laboratoire de Virologie, Nongo Conteya, BP 5680, Conakry, Guinea; 3. CD Eau Environnement, 2 rue de Belfays, 70190 Maizières, France; 4. Bernhard Nocht Institute for Tropical Medicine, Department of Virology, Bernhard-Nocht Strasse 74, 20359 Hamburg, Germany*

* mari-saeza@rki.de

Text S1

EBOLA INTERFERENCE

Guinea declared an EVD outbreak on 23 March 2014 in Gueckedou (Forest Guinea). As our first treatment finished in Faranah (Upper Guinea) in May 2014, the number of EVD clinical cases was 233, with 157 deaths [46] , and the epidemic had reached the capital, Conakry [47]. In August 2014, the president announced a state of emergency in Guinea, and most scientific and development projects were temporary interrupted. The first confirmed EVD case in Faranah was diagnosed in October 2014 [48]. The population, living in a difficult atmosphere, stopped going to health centres and hospitals. They also prevented foreigners from entering their villages, and they did not neither welcome well-known local team responsible for regular vaccinations.

When we resumed our intervention in Faranah (Upper Guinea) on 2 March 2015, after consultation with the province and district health authorities and local leaders, the local populations in 2 villages categorically refused to renew their collaboration with our research project. In 2 other villages, there were small areas with no acceptance, and in certain villages, we found bait stations in the garbage dumps behind huts. In general, during the EVD epidemic, the population in the 6 villages where the project operated remained sceptical towards research- and health-related activities.

Only after the end of the EVD epidemic, in August 2016, did the 2 villages that rejected the intervention activities request to re-establish their participation in the project. It was now clear to them that our project had no relationship with EVD. Fortunately, no EVD cases were recorded in the villages involved in the project intervention.

Interference with Ebola Outbreak

The EVD epidemic contributed to a disturbance in the perception of the intervention. A segment of the population was suspicious about the devices used for trapping (Sherman traps) and treatment (bait station), the necropsies of rodents and the collection of blood from healthy and sick people for epidemiological studies in our consortium. They were distrustful about the use of blood. During the EVD epidemic, some individuals suspected that our presence in Faranah was aimed at “sending them the disease”; others said later that some individuals complained about the "smell" from the bait station as if it was giving them a cold. However, in the same villages, other persons said that we were there before and after Ebola and that we were not related to the arrival of EVD in their locations. Certain individuals also mentioned that after 4 years, people now understood the intervention and have learned that rodents can give them diseases.
